# Genetics and Molecular Biology of Tuberous Sclerosis Complex

**DOI:** 10.2174/138920208786241243

**Published:** 2008-11

**Authors:** Valerio Napolioni, Paolo Curatolo

**Affiliations:** 1 Laboratory of Human Genetics, Department of Molecular, Cellular and Animal Biology, University of Camerino, Camerino, Italy; 2 Department of Neurosciences, Pediatric Neurology Unit, Tor Vergata University, Rome, Italy

**Keywords:** Tuberous sclerosis, tuberin, hamartin, mutations, genetics, multifactorial disease, germ-line mosaicism, rapamycin.

## Abstract

Tuberous Sclerosis Complex is a multisystem disorder exhibiting a wide range of manifestations characterized by tumour-like lesions called hamartomas in the brain, skin, eyes, heart, lungs and kidneys. Tuberous Sclerosis Complex is genetically determined with an autosomal dominant inheritance and is caused by inactivating mutations in either the TSC1 or TSC2 genes. TSC1/2 genes play a fundamental role in the regulation of phosphoinositide 3-kinase (PI3K) signalling pathway, inhibiting the mammalian target of rapamycin (mTOR) through activation of the GTPase activity of Rheb. Mutations in TSC1/2 genes impair the inhibitory function of the hamartin/tuberin complex, leading to phosphorylation of the downstream effectors of mTOR, p70 S6 kinase (S6K), ribosomal protein S6 and the elongation factor binding protein 4E-BP1, resulting in uncontrolled cell growth and tumourigenesis.

Despite recent promising genetic, diagnostic, and therapeutic advances in Tuberous Sclerosis Complex, continuing research in all aspects of this complex disease will be pivotal to decrease its associated morbidity and mortality. In this review we will discuss and analyse all the important findings in the molecular pathogenesis of Tuberous Sclerosis Complex, focusing on genetics and the molecular mechanisms that define this multisystemic disorder.

## INTRODUCTION

Tuberous Sclerosis Complex (TSC) is a dominantly inherited disease of high penetrance, characterized pathologically by the presence of hamartomas (tumour-like lesions) in multiple organ systems. Well known clinical manifestations include epilepsy, learning difficulties, behavioural problems, and skin lesions. Many patients have renal lesions, usually angiomyolipomas; cysts, polycystic renal disease, and renal carcinoma can also occur. Approximately one in 8,000 adults and one in 6,000 newborns are affected by TSC. Although TSC is often inherited, new mutations have been implicated in up to 75% of all cases. Males and females are equally likely to have TSC and the chance of passing it on to offspring is 50%.

Identification of the genes causing the condition and study of their protein products has shed light on the pathogenesis of the disease and provided valuable new information about signalling pathways regulating protein synthesis and cell growth. There is now the exciting possibility of drug therapy for some of the manifestations of the disease.

This review highlights the most significant concepts in the genetics and the molecular biology of TSC with emphasis on new advances in the knowledge of its pathophysiological mechanisms, the contribute given by animal models, and the role of rapamycin in TSC pharmacological therapy.

## CLINICAL OVERVIEW OF TUBEROUS SCLEROSIS COMPLEX

The clinical features of TSC have been reviewed in detail [1, 2]. Multisystem involvement in TSC results in a wide range of manifestations [3, 4].

### Neurological Phenotype

The commonest presentation is with seizures in infancy or early childhood, particularly infantile spasms. Partial and generalized seizures, atonic seizures (drop attacks) and myoclonic seizures also occur with the pattern of seizures evolving through childhood. A population-based study estimated that around 80% of children with TSC have epilepsy [5] and a prevalence of mental retardation of 44% which in two thirds of cases was profound (IQ<21). There is a strong association between mental retardation and epilepsy so that significant learning disability is very rare in patients with no history of seizures [5,6]. Risk factors for mental retardation include onset of seizures before 12 months of age, poor epilepsy control and infantile spasms. Infantile spasms develop in approximately one third of TSC patients and may be the initial symptom in almost 70% of affected infants that come to medical attention [7]. The majority of patients with TSC and infantile spasms unfortunately go on to develop other seizure types. Behaviour problems are common in TSC, particularly autism, autistic spectrum disorders, attention deficit hyperactivity disorder and sleep disturbance in children [8].

The common brain lesions in TSC are tubers in the cerebral cortex and subependymal nodules (SEN) along the lateral walls of the lateral ventricles [3]. Histologically, tubers are disorganized areas of cortex lacking the normal laminated architecture. Large cells resembling astrocytes but positive for both glial and neuronal markers are a conspicuous feature. SEN comprise a mixture of vascular stroma and astrocytic-like cells, some of which are large resembling those in tubers. Tubers may be associated with underlying white matter abnormalities such as migration lines [9]. Individuals with mental retardation tend to have more tubers than those with normal intelligence [6, 9] and it has been suggested that autism spectrum disorder is particularly associated with tubers in the temporal lobes [10]. SEN usually remain dormant throughout life, but they can increase in size, developing into a subependymal giant cell astrocytoma (SEGA) that occurs in 6–14% of TSC cases with the peak incidence in later childhood and adolescence.

### Renal Lesions

The renal manifestations of TSC include angiomyolipomas (AMLs), simple cysts, polycystic kidney disease, and renal-cell carcinoma. [11] These lesions likely arise in infancy or early childhood, increasing in size and number with age [12]. After neurologic complications, renal involvement is the second most common cause of morbidity and mortality in TSC [11]. AMLs are found in as many as 80% of TSC patients but are typically asymptomatic [11,12]. AML belongs to the family of perivascular epithelioid cell tumours and is typically composed of blood vessels, adipose tissue, and smooth muscle–like cells.

Renal cysts are also very common in TSC occurring in 17% of children and as many as 47% of adults. Like AMLs, they are frequently multiple and bilateral. However, renal cysts are more likely to become symptomatic than AMLs [11,12]. Polycystic kidney disease may also occur. It is a more severe, distinct entity with innumerable cysts that enlarge, replace renal parenchyma, and cause renal insufficiency and hypertension typically at an early age.

### Cardiac Lesions

Cardiac rhabdomyomas are benign tumours which are the most common childhood tumour involving the heart [3]. Cardiac rhabdomyomas are detected in ~60% of TSC patients and are often the first clinical manifestation of TSC [13]. Serial observations have demonstrated that the majority of these lesions become less prominent over time, with some disappearing altogether as assessed by ultrasound, so that surgical resection is performed only when they cause life-threatening complications. Pathologically, rhabdomyoma cells are aberrant glycogen-filled myocytes. Following routine histologic processing, loss of the glycogen leads to a distinctive appearance referred to as spider cells due to the radial arrangement of residual sarcoplasm extending out from the nucleus.

### Dermatological Phenotypes

Several types of skin lesion can occur in TSC [3]. Hypopigmented macules on the trunk and limbs are usually present at birth or become apparent during infancy. They can take any shape but are classically pointed at one end and rounded at the other resembling an ash leaf. Similar lesions on the scalp are associated with hypopigmented hair (poliosis). By 5 years of age, most children are developing angiofibromas on the face in the form of multiple flesh coloured or red papules. These typically occur over the nose, nasolabial folds, cheeks and chin. Fibrous plaques can develop on the forehead. Shagreen patches, which are raised brown or flesh coloured connective tissue naevi, often appear on the lower back during childhood. In adolescents and adults, ungual fibromas in the form of pink or red nodules on the finger and toe nails are a common finding. Gingival fibromas also occur. Some adults with TSC develop ‘confetti-like’ hypopigmentation on the limbs.

### Pulmonary Manifestations

Lymphangiomyomatosis (LAM) is a rare disorder of unknown aetiology caused by proliferation of atypical smooth muscle cells in the peribronchial, perivascular, and perilymphatic tissues of the lung [14]. LAM occurs almost exclusively in young women, typically presenting between 30 to 35 years of age. Rare cases of LAM have been reported in men, but the occurrence of LAM in men is controversial. LAM may occur sporadically but is also closely associated with TSC and it may affect up to 26% of female patients. Clinically, LAM is a progressive disorder of the lung, causing dyspnea, spontaneous pneumothorax, hemoptysis, cough, chylothorax cor pulmonale, and chest pain, eventually leading to progressive respiratory failure and death.

Histopathologically, LAM causes diffuse cystic destruction of the tissues of the lung by abnormal spindle-shaped, closely packed smooth-muscle cells. Multifocal, micronodular pneumocyte hyperplasia and clear-cell lung tumours have also been found in TSC patients. Renal AMLs are present in 50% of individuals with sporadic LAM and it has been hypothesized that LAM is the result of metastatic spread of benign AML smooth-muscle cells [14].

### Ophthalmologic Manifestations

The most common ocular findings in TSC are retinal hamartomas, appearing in 40% to 50% of patients [1, 15]. Their incidence increases with age. In the vast majority of TSC patients, they remain clinically stable and asymptomatic [16].

## GENETICS OF TUBEROUS SCLEROSIS COMPLEX

The prevalence of TSC is estimated at 1/10,000, and about two-thirds of the cases are sporadic with no family history due to new mutational events [17]; the mutation rate has been estimated at 2.5×10^−5^/gamete [18]. TSC is due to inactivating mutations in either of two genes, *TSC1 (on* chromosome 9q34) or *TSC2* (on chromosome 16p13.3). These mutations comprise the usual mix of nonsense, missense, insertion and deletion mutations, involving nearly all of the exons of *TSC1* and *TSC2 *(Table **1**). Although clinical expression of TSC varies greatly, in its classic form, there is 100% penetrance. Mutational studies of TSC patients have demonstrated that mutations in *TSC2* are about five times more common than mutations in *TSC1* in the sporadic TSC population, whereas the ratio is 1:1 in large families with multiple generations affected. Correspondingly, *TSC1* disease is milder than *TSC2* disease in multiple respects, which appears to be due to a reduced rate of second hit events [19-21]. Recently, several families have been described in which there are unusually mild manifestations of TSC, with most ‘affecteds’ not meeting diagnostic criteria, segregating with missense mutations in *TSC2* [22].

For many years, the prevailing model has been that the hamartomas of TSC develop through a two-hit mechanism in which there is complete loss of expression of functional *TSC1* or *TSC2*, supported by findings of loss of heterozygosity (LOH) in TSC tumour samples [23-26]. However, the rate of LOH appears to vary according to lesion type, and LOH may not occur in all SEGAs and cortical tubers [24, 26]. On the other hand, recent evidence suggests that both tuber giant cells and SEGA cells have similar immunophenotypes, and SEGAs commonly sustain two-hit inactivation of either *TSC1* or *TSC2*.

### Structure of Hamartin (TSC1) and Tuberin (TSC2) 

The TSC genes *TSC1* and *TSC2* were first identified by positional cloning strategies. They encode previously unknown proteins, termed hamartin and tuberin respectively, that form a functional complex.

The *TSC1* gene consists of 23 exons, of which the last 21 contain coding sequence and the second is alternatively spliced [27]. Maximal promoter activity is present in a 587-bp region + 77 to -510 bp with respect to the transcription start site (TSS) in the TSC1 upstream region. Interestingly, this region contains no consensus TATA box or CAAT box. However, a 521-bp fragment surrounding the TSS exhibits the characteristics of a CpG island which overlaps with the promoter region. Putative binding sites for several known transcription factors, namely Sp1, E2F, CdxA, GATA, c-Ets, HSF2, Ik2, USF and SRY are found in the upstream region [28].

**Fig. (1). Biochemical structure of hamartin and tuberin.**
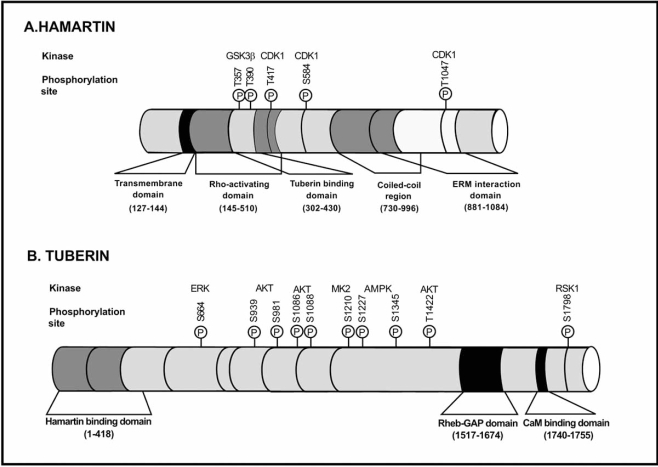
In this figure are rappresented the regulatory phosphorylation sites and respective kinases responsible for their phosphorylation.

Hamartin, the predicted product, comprised 1164 amino acids (130 kDa). It had no significant homology to tuberin or other known vertebrate proteins but did have significant homology to a *Schizosaccharomyces pombe *predicted protein [29]. Hamartin is ubiquitously expressed [27], and contains a putative transmembrane domain at amino acids 127–144 and a coiled coil domain (CCD) spanning amino acids 719–998 [30]. The amino acid residues 145–510 of hamartin contain the function for activation of Rho GTPase, and amino acid residues 881–1084 interact with the N-terminal of the ezrin–radixin–moezin (ERM) family of actin-binding proteins (Fig. **1**) [31, 32]. These ubiquitously expressed proteins crosslink cortical actin filaments to the plasma membrane, organising the cytoskeleton and acting as substrates for the tyrosine kinase of the epidermal growth factor receptor. Hamartin has also been shown to interact with neurofilament-L [33].

The TSC2 gene has 41 small exons spanning 45 kb of genomic DNA and encodes a 5.5 kb mRNA. Exons 25, 26 and 31 are subject to alternative splicing [17]. The encoded protein tuberin has a full-length isoform of 1807 amino acids (198 kDa). A region spanning residues 1517–1674 and encoded by exons 34–38 has significant homology to the GTPase-activiating proteins (‘GAPs’) human rap1 GAP and murine Spa1 (Fig. **1**) [30,34]. However, only modest GAP activity of tuberin for rap1 and rab5 has been demonstrated biochemically.

*TSC2* contains a calmodulin-binding domain and an oestrogen-receptor-a-binding domain [35]. 

The gene for polycystic kidney disease, *PKD1*, is located immediately centromeric to *TSC2*, accounting for the occurrence of both conditions in families with large rearrangements (contiguous gene syndromes) [36]. The C-terminal domain within the *TSC2* protein was recognized as being homologous to other GAP domains when it was cloned in 1993 [30].

*TSC1* and *TSC2* regions responsible for heterodimerisation have been identified [37]. The region of hamartin known to span the interacting domain with tuberin is within the amino acids 302–430, and the first 418 amino acids of tuberin contain the binding site for hamartin. Hamartin stabilizes tuberin by inhibiting its interaction with the HERC1 ubiquitin ligase [38]. Proteasome-mediated degradation of tuberin has been shown to be induced *via *its binding to the E6 oncoprotein of the human papillomavirus (HPV16 E6). HPV16 E6-induced degradation of tuberin leads to activation of S6K [39]. Binding of FIP200 to hamartin was suggested to disrupt the TSC protein complex formation [40]. Another hamartin binding protein has also been implicated in the regulation of the TSC tumour suppressor complex. TBC7 was reported to enhance ubiquitination and degradation of hamartin [41].

The two chaperone proteins, HSCP-70 and HSP70-1, bind to tuberin. Chaperone proteins recognise and bind misfolded proteins. Increased interactions of both chaperone proteins with the disease causing tuberin mutant R611Q have been reported [42]. The hamartin/tuberin heterodimeric complex formation provides a tentative explanation for the similar disease phenotype in TSC patients with mutations in either of the two TSC genes.

Hamartin and tuberin are coexpressed in cells of several organs, such as kidney, brain, lung, and pancreas. Hamartin has been reported to interact with Plk1 and to be localized to the centrosome. Cdk1 phosphorylates hamartin at several sites, of which the phosphorylation at T310 regulates its interaction with Plk1 [43].

Tuberin has been described to be localised to the cytosol and the membrane fraction within the cytoplasm and to the nucleus. Akt-mediated phosphorylation of tuberin has been demonstrated to regulate both, the translocation of tuberin from the membrane to cytosol and the nuclear/cytoplasmic localisation of tuberin [44].

### Mutations and Polymorphisms

The mutation spectra of the TSC genes are very heterogeneous and no hotspots for mutations have been reported. Indeed, more than 200 *TSC1* and 700 *TSC2 *unique allelic variants have been reported [20, 21, 45-48]. There are many mutations in each gene that are seen recurrently, but no single mutation accounts for more than about 1% of all TSC patients. Despite complete penetrance of the disease, phenotypic variability can make the determination of disease status difficult among family members of affected individuals.

**Fig. (2):**
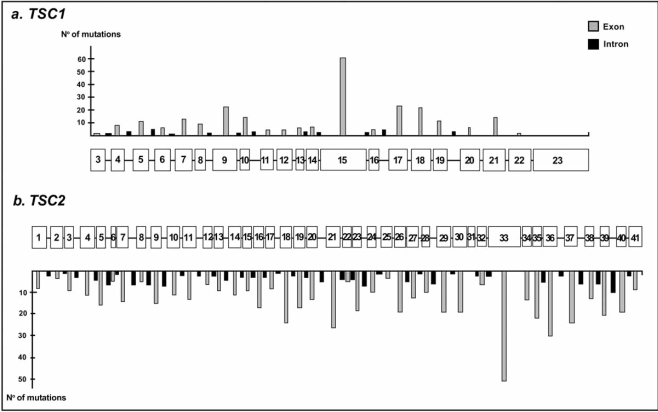
Mutation spectra of *TSC1* and *TSC2*.

The major proportions in both genes are subtle mutations composed of nonsense mutations, small deletions and insertions, splice site changes, and, for *TSC2*, additionally missense mutations. All these mutations are distributed over the entire regions of both genes (Fig. **2**).

Among the large number of *TSC* mutations identified as disease causing, only a few affect splicing regulatory sites. The frequency and nature of *TSC* splice site mutations reported so far are in agreement with these overall findings: in *TSC1* they represent 8% and in *TSC2* they comprise 12% of the total number of identified mutations. Most changes were merely assumed to influence the mature transcripts, especially when they affect highly conserved sequences at exon/intron junctions, while few are confirmed on the transcript or the protein level. Mayer *et al*. [49] performed an RNA based screening of the entire coding regions of both *TSC* genes applying the protein truncation test (PTT) and identified a high proportion of unusual splicing abnormalities affecting the *TSC2* gene. Two cases exhibited different splice acceptor mutations in intron 9 (IVS9315GCA and IVS933CCG) both accompanied by exon 10 skipping and simultaneous usage of a cryptic splice acceptor in exon 10. Another splice acceptor mutation (IVS38318ACG) destroyed the putative polypyrimidine structure in intron 38 and resulted in simultaneous intron retention and usage of a downstream cryptic splice acceptor in exon 39. Another patient bore a CCT transition in intron 8 (IVS8+281CCT) activating a splice donor site and resulting in the inclusion of a newly recognised exon in the mRNA followed by a premature stop.

Nellist *et al*. [50] showed that pathogenic tuberin amino-acid substitutions disrupt the tuberin–hamartin complex and subsequently have investigated how these mutations affect the role of tuberin in the control of signal transduction through mTOR. The R611Q, R611W, A614D, C696Y and V769E substitutions [51] disrupted the tuberin–hamartin interaction, and prevented the phosphorylation of tuberin by *PKB*, the inhibition of S6 and S6K phosphorylation, and the stimulation of Rheb GTPase activity, cause TSC because they result in major conformational changes to tuberin. The 609insS and F615S amino-acid changes play a similar effect, but do not completely inactivate tuberin.

The N525S and K599M substitutions inhibited the phosphorylation of S6K and S6, and increased the GTPase activity of Rheb. The K599M substitution is a de novo mutation [46] and has been shown to reduce the tuberin–dependent inhibition of phosphorylation of overexpressed 4E-BP1 [52]. However, this effect is weak compared to the V769E tuberin variant [53].

Recently, Nellist *et al*. [54] found that deletion of isoleucine at amino acid residue 820 of TSC2 and the TSC2 L1511H, C244R and Y598H amino acid substitutions are sufficient to cause TSC. The TSC2 R1772C, T993M, S132C, F143L and A196T substitutions are rare polymorphisms that do not inhibit TSC1–TSC2 function, and do not cause TSC.

Previous reports have identified a mutation consisting of a 34 bp deletion affecting portions of exon 38 and the adjacent intron 38 of *TSC2*. Roberts *et al*. [55] found this genetic variation in 4 of 800 TSC patients. In every case, the variant was present in one unaffected parent of the sporadically affected child. They excluded the possibility of mosaicism in the parents with this variant and conclude that this deletion is a rare polymorphism that does not cause TSC, but may be a modifier of the TSC phenotype.

Major genes for TSC and autosomal dominant polycystic kidney disease (PKD), *TSC2* and *PKD1*, respectively, lie adjacent to each other at chromosome 16p3.3, suggesting a role for *PKD1 *in the etiology of renal cystic disease in TSC.

Features of TSC and autosomal dominant PKD have been observed in patients with a *TSC2-PKD1 *contiguous gene syndrome. In these patients a large part of the adjacent *TSC2 *and *PKD1 *genes has been deleted on one chromosome. In a study described by Sampson *et al*. [56] 17 of 22 patients with such a deletion have been diagnosed with a very severe form of PKD, already manifesting within the first year of life. A possible explanation for this severe phenotype is a functional link between the *TSC2* protein and polycystin-1 in protein sorting as described by Kleymenova *et al*. [57]. The frequency of genomic deletions involving only the *PKD1*-gene is low [58]. Ariyurek *et al*. [58] observed 4 deletions in 125 patients. In another study a 5-kb deletion (region intron 34-3'UTR) and a 2-kb deletion (region intron 30-34) were found upon analysis in a set of 167 patients [59]. Furthermore, a 3-kb deletion was reported in the region intron 1-exon 5, identified using long-range PCR performed on 24 patients [60]. Likely, patients with a deletion that disrupts the *PKD1 *and *TSC2* genes are usually identified as TSC patients. Mosaicism for deletions involving *TSC2* and *PKD1* was a frequent phenomenon and was associated with preserved renal function in some cases. Among mosaics, disease severity did not correlate with the frequency of the mutant allele in lymphocytes; the level of mosaicism in renal tissue is likely to be more important. Five of the 27 unrelated patients studied by Sampson *et al*. [56] had multiple cysts in both kidneys, but no detectable disruption of *PKD1*. All were identified, through ultrasound screening, as having renal cystic disease. Large rearrangements of *TSC2* were defined in 3 of these patients.

The high degree of variability of TSC clinical manifestations, including those among related and unrelated patients with the same mutation [19], suggests the possibility that modifier genes influence disease severity.

Because *IFN-γ* has been shown to be a useful mediator of tumour regression in animal models of kidney tumours [61, 62] and because there is a known high-expressing allele of *IFN-γ* in humans, Dabora *et al*. [63] examined the relationship between the *IFN-γ* genotype and the severity of renal disease in TSC patients who had *TSC2 *mutations; they found an association between *IFN-γ* allele 2 and the absence of kidney AMLs in *TSC2 *patients.

This finding suggests that *IFN-γ* allele 2 may be a genetic modifier that reduces kidney AMLs development or growth. Because allele 2 has been shown to be associated with a higher level of *IFN-γ *expression in mitogen-stimulated mononuclear cells *in vitro* [64], it is plausible that this association is due to a reduction in kidney AMLs development in the presence of higher levels of *IFN-γ*.

The enzyme 8-oxoguanine glycosylase 1 (*OGG1*) repairs 8-oxo-2-deoxyguanosine residue (8-oxodG) an oxidatively damaged promutagenic base. Genetic variations in *OGG1* gene have been shown to modulate DNA repair capacity and are related risk of tumour development. Habib *et al*. [65] tried to determine whether genetic variants in *OGG1* play a role in susceptibility to AML in TSC patients. They identified showed the presence of significant association between the Ser326Cys polymorphism of *OGG1* and AML. Moreover, they also assessed the presence of oxidative DNA damage in kidney sections by immunostaining for 8-oxodG. 8-OxodG staining was highly abundant in kidney AML tissue from TSC patients compared to weak staining in uninvolved tissue from the same TSC patients or normal kidney from healthy subjects. Taken together, these findings suggest that *OGG1 *Ser326Cys variant of may confer risk for development of AMLs by increasing oxidative DNA damage.

Following the observation of a TSC patient with a de novo reciprocal translocation t(3;12) (p26.3;q23.3), Fahsold *et al*. [66] have undertaken a linkage study in 15 TSC families using polymorphic DNA markers neighbouring the chromosome breakpoints. Significant lod scores have been obtained for markers *D12S7* (Zmax = 2.34, theta = 0.14) and *PAH* (phenylalanine hydroxylase) (Zmax = 4.34, theta = 0.0). In multipoint linkage analysis, the peak lod score was 4.56 at the *PAH* gene locus. These data suggest the existence of a third gene locus for TSC (*TSC3*) on chromosome 12q22-24.1. Dysfunctions of phenylalanine hydroxylase pathway might be involved in the pathogenesis of TSC.

### Germ-Line Mosaicism Hypothesis

Germ-line mosaicism has been demonstrated in both common and uncommon genetic disorders [67]. Because most germ-line mutations are likely to be mitotic in origin and because the mutation rate multiplied by the number of mitoses necessary to form the gametes is 11 [68], germ-line mosaicism would be expected to occur to some degree in all genetic disorders. Empiric recurrence risks of specific diseases can be estimated, but risks for individual families depend on the percentage of affected gametes in the germ line of the parent with mosaicism.

Fifteen families with two or more affected children and apparently unaffected parents have been reported and are thought to illustrate examples of germ-line mosaicism [69, 70]. Yates *et al*. [71] have proved germ-line mosaicism for *TSC2* in one family, by molecular analysis.

Unaffected parents who have had a child affected with TSC usually are given a low (1%) recurrence risk. Evidence that germ-line mosaicism is not an uncommon phenomenon would increase the estimated risk in cases of sporadic TSC and, thus, would have implications for genetic counselling.

Rose *et al*. [72] found germ-line mosaicism in five families with mutations in the *TSC2* gene and in one family with the causative mutation in the *TSC1* gene.

## MOLECULAR BIOLOGY OF TUBEROUS SCLEROSIS COMPLEX

Led by seminal studies in Drosophila, the *TSC1/TSC2* complex has been positioned in an ancestrally conserved signalling pathway that regulates cell growth. *TSC1/TSC2* receives inputs from at least three major signalling pathways in the form of kinase-mediated phosphorylation events that regulate its function as a GAP protein [73]: the PI3K-Akt pathway, the ERK1/2-RSK1 pathway and the LKB1-AMPK pathway.

**Fig. (3). Tuberous sclerosis complex signalling.**
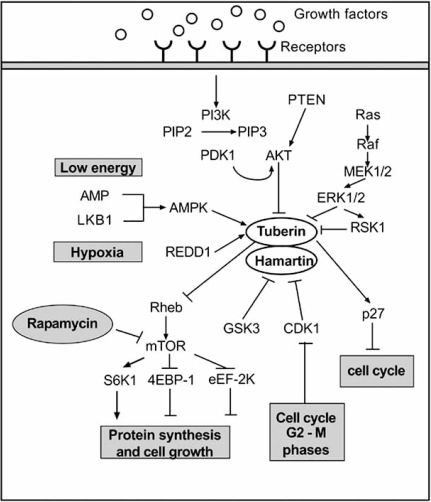
Figure showing signalling pathways involved in the regulation of TSC complex controlling mammalian target of rapamycin (mTOR) activity. PI3K=phosphatidylinositol 3-kinase. PIP2=phosphatidyl-inositol (4,5) biphosphate. PIP3= phosphatidyl-inositol (3,4,5) triphosphate. PDK1= phosphoinositide-dependent protein kinase 1. PTEN= Phosphatase and tensin homolog. AKT=Protein kinase B. REDD-1=DNA-damage inducible transcript 4 protein. RSK-1=ribosomal protein S6 kinase alpha-1. LKB1=Serine/threonine-protein kinase 11. ERK=extracellular signal-related kinase. Rheb=Ras homologue enriched in brain. S6K1=ribosomal protein S6 kinase beta-1. 4E-BP1=eukaryotic translation initiation factor 4E-binding protein 1. eEF-2K=elongation factor 2 kinase. CDK1= Cyclin dependent Kinase 1.

*TSC1/TSC2* functions as a GAP towards Rheb, which is a major regulator of the mammalian target of rapamycin (mTOR). In the absence of either *TSC1* or *TSC2*, high levels of Rheb-GTP lead to constitutive activation of mTOR–raptor signalling, thereby leading to enhanced and deregulated protein synthesis and cell growth (Fig. **3**).

### TSC Upstream Pathways 

The pathways signalling through *TSC1–2* have multiple separate phosphorylation sites. Sites identified so far include those for GSK3β (glycogen synthase kinase 3β) [74] and CDK1 (cyclin dependent kinase 1) [75] on *TSC1* and those for ERK1/2 (extracellular signal-regulated kinase 1 and 2) and RSK1 (p90 ribosomal protein S6 kinase 1) [76-78], MK2 [79], Akt (protein kinase B) [80], AMPK (adenosine monophosphate-activated protein kinase) [81] and GSK3β [82] on *TSC2*.

Inoki *et al*. [82] suggested a possible function of *TSC2*/mTOR signalling in tumourigenesis caused by dysfunction of the Wnt pathway and a mechanism by which Wnt stimulates protein synthesis and cell growth. This possibility is given by the fact that Wnt stimulates the mTOR signalling pathway *via *inhibiting GSK3 phosphorylation of TSC2. 

The phosphorylation of tuberin by Akt and MK2 promotes the binding of tuberin with 14-3-3 proteins. 14-3-3 proteins are members of a group of proteins that specifically interact with phosphorylated proteins, facilitating the phosphorylation-dependent control of protein activity [83]. Detection of a ternary complex of tuberin, hamartin and 14-3-3 suggests that the tuberin-14-3-3 interaction is compatible with tuberin-hamartin binding and that 14-3-3 proteins interact with the tuberin-hamartin complex [84, 85]. A possible function of the interaction between 14-3-3 proteins and phosphorylated tuberin is to inhibit the formation of tuberin-hamartin complex, in order to decrease the stability of tuberin and release of the activated mTOR [84].

Recently, Yasui *et al*. [86] identified NADE (p75NTR-associated cell death executor) as a novel interactor protein with hamartin. NADE has been shown to mediate NGF-induced apoptosis in neuronal cells through the interaction with p75NTR [87, 88]. Hamartin binds to NADE with its coiled coil domain domain. Down-regulation of hamartin with *TSC1* siRNA led to failure of NGF-induced apoptosis in PC12h cells suggesting that the association of hamartin with NADE is involved in neuronal cell death, which could explain why hamartoma cells are not eliminated in TSC. Current data supports a model that, apart from receptor tyrosine kinases, G protein-coupled receptors are also able to regulate tuberin activity [89]. Direct evidence has shown that G i/o - and G q -coupled receptors can regulate tuberin phosphorylation in a PI3K-dependent or –independent manner, while G 12/13 may have a dual role in regulating tuberin.

A recent study has demonstrated that the forkhead transcription factor FoxO is capable of binding to tuberin [90]. FoxO binds to an adjacent region near the GAP domain, thus inhibiting the GAP activity towards Rheb.

### TSC Downstream Pathways 

The best understood output pathway is from the GAP domain of *TSC2*, although other downstream pathways are also likely.

Phosphorylation of several sites on *TSC1–2* stimulates the GAP function of *TSC2*, whereas phosphorylation of other sites inhibits it [37, 73, 91, 92]. Inhibition of the GAP function of *TSC2* shifts the balance of its substrate Rheb to the Rheb-GTP form, which activates the mTOR protein and ultimately leads to phosphorylation of ribosomal protein S6 and 4E-BP1 resulting in increased protein synthesis and cell proliferation. *TSC1–2* is also proposed to function in cell-cycle control by regulating the cyclin-dependent kinase inhibitor p27 [93]. p27 protein levels are regulated through ubiquitin-dependent degradation [94].

Skp2 is the F-box protein, which together with other proteins forms an SCF-type E3 ubiquitin ligase complex, whose task is to target p27 for degradation by the proteasome [95, 96]. Neither tuberin nor hamartin are in a complex with Skp2 and tuberin does not affect Skp2 protein levels, and the SCFSkp2 ubiquitin ligase does not regulate tuberin stability. However, binding of tuberin to p27 sequesters p27 from Skp2 accompanied by a stabilization of the p27 interaction with cdk2, and hence, Skp2-induced p27 degradation and cell cycle progression is abolished by tuberin’s protective binding to p27. The observed binding of the tumour suppressor protein tuberin to the tumour suppressor protein p27 provides a molecular explanation for the effects of the TSC genes on p27 protein stability [97]. Through Rac and Rho, *TSC1* and *TSC2* have a signalling role in the development and maintenance of the actin cytoskeleton [31, 32].

Ozcan *et al*. [98] found that loss of TSC1 or TSC2 in cell lines and mouse or human tumours caused endoplasmic reticulum (ER) stress and activated the unfolded protein response. The resulting ER stress played a significant role in the mTOR-mediated negative feedback inhibition of insulin action and increased the vulnerability to apoptosis

Tuberin downregulates the DNA repair enzyme 8-oxoguanine DNA-glycosylase (OGG1) with important functional consequences, compromising the ability of cells to repair damaged DNA resulting in the accumulation of the mutagenic oxidized DNA, 8-oxo-dG. OGG1 localizes with tuberin preferentially in kidney cortex. Loss of tuberin is accompanied by the loss of OGG1 contributing to tumourgenesis [99].

## ANIMAL MODELS OF TUBEROUS SCLEROSIS

Mouse and *Drosophila* models carrying mutant *TSC1* and *TSC2* alleles have been reported, as has a naturally occurring *TSC2* mutant rat, the ‘Eker’ rat. These models have provided a valuable resource for investigating the functions of *TSC1* and *TSC2*.

### Eker Rat

First described in the 1950s, the Eker rat strain contains a germline inactivation of one allele of the gene encoding *TSC2* and has served as an animal model for hereditary renal cell carcinoma [100, 101]. Following the identification of *TSC1* and *TSC2* as genes associated with human disease, murine models lacking *TSC1* or *TSC2* were generated by gene targeting [102].

In these models, homozygous *TSC1* or *TSC2* mutants die at an embryonic stage, whereas heterozygous carriers are predisposed to tumour formation. These studies confirmed the tumour suppressor function of *TSC1* and *TSC2* as inferred from human genetic analysis of the TSC.

In particular, the Eker rat carries an inactivating retrotransposon insertion mutation in exon 30 of the *TSC2 *gene [103]. It develops bilateral multifocal solid and cystic renal adenomas and extra-renal pathology, including uterine leiomyoma, splenic haemangioma, pituitary adenoma and SEN hamartomas in the brain [104]. The phenotype is transmitted as an autosomal dominant trait with embryonic lethality in the homozygote at days 10–12 with disrupted neuroepithelial growth and development [101]. Allelic loss and intragenic mutation have been demonstrated in tumours in the Eker rat [105] and transgenic expression of *TSC2 *in the Eker rat and Eker tumour cell lines support its tumour-suppressor function [100].

### TSC1- and TSC2-Knockout Mice

Mice with targeted disruption of *TSC1* or *TSC2* have been generated and express a phenotype similar to the Eker rat [102]. Multiple renal cystadenomas develop and lead to renal cell carcinoma in approx. 5–15% of mice by 18–24 months. Although there is considerable inter-strain variability in tumour development this appears to be more rapid in *TSC2* than *TSC1* heterozygotes. Histologically benign hepatic haemangiomas and histologically malignant but non-metastasizing haemangiosarcomas of the limbs and tail also occur. Lung adenomas have also been observed, but seem to have limited growth potential. Homozygotes die in midgestation (E10.5– E12.5) and exhibit growth failure, hepatic hypoplasia, cardiac hypertrophy and anaemia.

### Drosophila

The identification of *TSC1* and *TSC2* homologues in *Drosophila* coincided with an exciting burst of studies that implicate a role for the insulin signalling pathway in the control of cell growth in *Drosophila* and mammals. Cell growth – the process in which cells accumulate mass – is a distinct process, which should be distinguished from cell proliferation, because cell proliferation per se does not necessarily drive cell growth [106].

The initial implication of insulin signalling in the control of cell size in *Drosophila* was demonstrated by the fact that PI3K (p110) and IRS (chico) are required cell-autonomously to promotes cell growth [107, 108]; subsequently, various studies showed the involvement of other components of the insulin pathway in cell growth, including Akt [109], PTEN [110], PDK1 [111] and S6K [112]. Collectively, these studies demonstrate that downregulation of the insulin pathway leads to decreased cell size, whereas upregulation of this pathway results in the opposite phenotype. A unique feature of the Drosophila system is that, in this organism, it is possible to monitor cell growth in ‘mosaic’ animals containing genetically mutant cells in an otherwise wildtype genetic background; this allows an investigation of whether a given gene is required cell-autonomously or non cell-autonomously for cell growth.

Homozygous mutations of the *TSC2 *gene are lethal to *Drosophila *during larval development. However, *TSC2*−*/*− clones generated in specific tissues exhibit the ‘gigas’ phenotype characterized by increased cell and organ size with generally normal differentiation and morphology [113]. Mutagenesis screens revealed that *TSC1 *mutations also cause a gigas-like phenotype [114, 115].

### Schizosaccharomyces Pombe

Extensive homology search fails to identify *TSC1* and *TSC2* homologues in the worm *Caenorhabditis elegans* or the budding yeast *Saccharomyces Cerevisiae* [116]. At present, it is unclear whether this reflects a true absence of such molecules or functional homologues of *TSC1* and *TSC2*, in these organisms, have diverged beyond recognition by simple homology search. By contrast, the fission yeast *Schizosaccharomyces pombe* contains possible homologues of *TSC1* and *TSC2* [116]. Similar to their mammalian counterparts, the *TSC1* and *TSC2* homologues of *S. pombe* form a protein complex. Deletion of the *TSC1* or *TSC2* genes in this organism results in similar defects in the localization of amino acid permease, nutrient uptake and conjugation. Although studies of *TSC1* and *TSC2* homologues in *S. pombe* are at an early stage, because of the simplicity of this organism and the power of genetic analysis, this system clearly has tremendous potential as a genetic model for understanding the molecular mechanisms of *TSC1* and *TSC2* functions.

## RAPAMYCIN: MORE THAN A PROMISE

Rapamycin, also referred to as Sirolimus, has powerful antiproliferative and immunosuppressant activity [117]. Since 1999, it has been United States Food and Drug Administration approved for the prophylaxis of organ rejection in patients older than 13 years. Rapamycin, discovered in 1965 from soil samples on Easter Island, known to its indigenous population as Rapa Nui, was isolated from *Streptomyces hygroscopicus* [117]. It was found to be active against several strains of yeast and fungi. However, no activity was observed against gram-positive or gram-negative bacteria.

### Rapamycin and Tuberous Sclerosis Complex

Studies on sirolimus activity have shown that it binds to its intracellular receptor FKBP12 (FK506-binding protein 12), a member of the family of FK506-binding proteins. It has also been demonstrated that the binding of sirolimus to FKBP12 is required for the inhibitory effect of sirolimus on mTOR function [118]. It has been shown that the sirolimus/FKBP12 complex, but not FK506/FKBP12 complex, binds with very high affinity to mTOR. At the same time, FK506 competitively inhibits the effects of sirolimus that are mediated by mTOR. Unlike most kinase inhibitors, sirolimus does not completely inhibit the kinase activity of mTOR [119]. Instead, sirolimus/FKBP12 complex activity causes derepression of specific protein phosphatases, which leads to dephosphorylation of mTOR downstream effectors like S6K1 or 4E-BP1. Therefore, Rapamycin, increasing levels of unphosphorylated S6K, causes inhibition of translation, and produces cell cycle arrest and the possibility of reduced hamartoma formation or regression [120].

In addition, many of the skin, brain, and kidney hamartomas seen in TSC, including cutaneous angiofibromas and LAM, are vascular and contain an endothelial component.

Sirolimus decreased VEGF production, which may also be beneficial in these vascular tumours because of its antiangiogenic effects [117, 120]. Oral sirolimus therapy can also induce regression of astrocytomas associated with TSC. Five patients with TSC and astrocytomas were treated with oral sirolimus at standard immunosuppressive doses (serum levels 5-15 ng/mL) from 2.5 to 20 months and showed astrocytoma regression [121]. An open-label clinical trial demonstrated that AMLs in TSC patients regressed somewhat during sirolimus therapy but tended to increase in volume after the therapy was stopped [122]. Rauktys *et al*. [123] have also shown that topical administration of rapamycin is an effective treatment for TSC-related tumours in a mouse model demonstrating that transdermal delivery of rapamycin is feasible and topical rapamycin should be further investigated as a novel treatment approach for TSC skin disease such as facial angiofibromas.

If one can establish the prenatal diagnosis of TSC and begin using sirolimus early, it may be possible to prevent the development of TSC manifestations, similar to the situation with early diagnosed and treated phenylketonuria [124]. However, the utility of mTOR inhibitors in treating epilepsy in TSC has not been investigated in either clinical trials or animal models. Zeng *et al*. [125] recently demonstrated that rapamycin has strong efficacy for preventing seizures and prolonging survival in *Tsc1*GFAPCKO mice, a mouse model of TSC with conditional inactivation of the Tsc1 gene in glial fibrillary acidic protein (GFAP)–positive cells (Tsc1GFAPCKO mice), which develops progressive epilepsy, encephalopathy, and premature death, as well as cellular and molecular brain abnormalities likely contributing to epileptogenesis.

## CONCLUSION/FUTURE DIRECTIONS

Significant progress has been made in the understanding of the genetic and pathogenic aspects of this serious and multi-system disorder. A multidisciplinary approach is essential for an early, accurate diagnosis and proper management of affected individuals. The genetic basis of TSC is now well understood and genetic testing is available for the majority of families, but better methods need to be developed for rapid and reliable identification of pathogenic mutations. Key priorities for future research include a better understanding of functional relationship between TSC1 and TSC2 and their pathways and a gained insight into the relationships between genotypes and phenotypes. 

The delineation of the TSC biochemical signalling pathways suggest strategies for developing targeted therapies including mTOR inhibition, which are being evaluated in clinical trials.

## Figures and Tables

**Table 1.** Number of Mutation Reported for Tuberous Sclerosis Complex Patients

Mutation	TSC1	Percentage	TSC2	Percentage
Substitutions	97	36.3%	380	49.4%
Insertions	10	3.7%	36	4.7%
Deletion	117	43.8%	265	34.5%
Duplication	41	15.4%	80	10.4%
Insertion/deletions	2	0.8%	8	1.0%
Total	267		769	
